# Investigating the association between fasting insulin, erythrocytosis and HbA1c through Mendelian randomization and observational analyses

**DOI:** 10.3389/fendo.2023.1146099

**Published:** 2023-03-17

**Authors:** Anthony Nguyen, Rana Khafagy, Habiba Hashemy, Kevin H. M. Kuo, Delnaz Roshandel, Andrew D. Paterson, Satya Dash

**Affiliations:** ^1^ Department of Medicine, University Health Network, University of Toronto, Toronto, ON, Canada; ^2^ Genetics and Genome Biology Program, The Hospital for Sick Children, Toronto, ON, Canada; ^3^ Divisions of Epidemiology and Biostatistics, Dalla Lana School of Public Health, University of Toronto, Toronto, ON, Canada; ^4^ Division of Medical Oncology and Haematology, Department of Medicine, University Health Network, Toronto, ON, Canada; ^5^ Division of Haematology, Department of Medicine, University of Toronto, Toronto, ON, Canada; ^6^ Institute of Health Policy, Management and Evaluation, Dalla Lana School of Public Health, University of Toronto, Toronto, ON, Canada

**Keywords:** insulin resistance, hyperinsulinemia, type 2 diabetes (T2D), hemoglobin A1c, erythrocytosis

## Abstract

**Background:**

Insulin resistance (IR) with associated compensatory hyperinsulinemia (HI) are early abnormalities in the etiology of prediabetes (preT2D) and type 2 diabetes (T2D). IR and HI also associate with increased erythrocytosis. Hemoglobin A1c (HbA1c) is commonly used to diagnose and monitor preT2D and T2D, but can be influenced by erythrocytosis independent of glycemia.

**Methods:**

We undertook bidirectional Mendelian randomization (MR) in individuals of European ancestry to investigate potential causal associations between increased fasting insulin adjusted for BMI (FI), erythrocytosis and its non-glycemic impact on HbA1c. We investigated the association between the triglyceride-glucose index (TGI), a surrogate measure of IR and HI, and glycation gap (difference between measured HbA1c and predicted HbA1c derived from linear regression of fasting glucose) in people with normoglycemia and preT2D.

**Results:**

Inverse variance weighted MR (IVWMR) suggested that increased FI increases hemoglobin (Hb, b=0.54 ± 0.09, p=2.7 x 10^-10^), red cell count (RCC, b=0.54 ± 0.12, p=5.38x10^-6^) and reticulocyte (RETIC, b=0.70 ± 0.15, p=2.18x10^-6^). Multivariable MR indicated that increased FI did not impact HbA1c (b=0.23 ± 0.16, p=0.162) but reduced HbA1c after adjustment for T2D (b=0.31 ± 0.13, p=0.016). Increased Hb (b=0.03 ± 0.01, p=0.02), RCC (b=0.02 ± 0.01, p=0.04) and RETIC (b=0.03 ± 0.01, p=0.002) might modestly increase FI. In the observational cohort, increased TGI associated with decreased glycation gap, (i.e., measured HbA1c was lower than expected based on fasting glucose, (b=-0.09 ± 0.009, p<0.0001)) in people with preT2D but not in those with normoglycemia (b=0.02 ± 0.007, p<0.0001).

**Conclusions:**

MR suggests increased FI increases erythrocytosis and might potentially decrease HbA1c by non-glycemic effects. Increased TGI, a surrogate measure of increased FI, associates with lower-than-expected HbA1c in people with preT2D. These findings merit confirmatory studies to evaluate their clinical significance.

## Introduction

The type 2 diabetes (T2D) pandemic is a major public health challenge, affecting more than 420 million people worldwide (1, 2). Insulin resistance (IR) and associated compensatory hyperinsulinemia (HI) are early abnormalities in the pathogenesis of prediabetes (preT2D) and Type 2 diabetes (T2D) (3). Although reduced insulin action (IR) is implicated in hyperglycemia, some aspects of insulin signaling pathways are preserved in states of IR. Consequently, some manifestations associated with IR are due to HI (4, 5). Close surveillance and timely intervention in people with IR and HI can potentially prevent T2D and remit/improve glycemia in those who develop T2D (6–8).

Increasingly, hemoglobin A1c (HbA1c) has replaced fasting glucose and/or the 75 g oral glucose tolerance test to diagnose preT2D, T2D and T2D remission. HbA1c is also used to set glycemic targets for people with diabetes (9–11). Advantages to using HbA1c compared to fasting glucose include convenience and use of an assay that is standardized, stable and reproducible with limited intraindividual variability. Further, it provides an average measure of glycemia in the prior 2 to 3 months (1, 12) (1). However, altered red cell lifespan and erythrocytosis can affect HbA1c measurement by non-glycemic pathways. This has implications in patients with red cell disorders and hemoglobinopathies (1, 13). In people without T2D, including those with preT2D, non-glycemic parameters are a major predictor of HbA1c. Higher hemoglobin (Hb) associates with lower HbA1c (14, 15). Observational studies have also reported higher Hb and red cell count with increased IR and HI (16–18). Whether this association is causal is not established nor is it known if it impacts HbA1c measurement through non-glycemic pathways. HI can potentially increase cell proliferation and thus plausibly mediate the increased erythrocytosis seen in people with IR and HI (19).

Mendelian randomization (MR) can be used to infer potential causal associations between an exposure and an outcome by assessing the effects of genetic variants robustly associated with the exposure in one population on the outcome of interest in a separate cohort (2 sample MR) (20). We undertook bidirectional MR to investigate potential causal associations between fasting insulin adjusted for BMI (FI) and measures of erythrocytosis (hemoglobin, measured as g/L of blood, red cell count and reticulocyte count) in people of European ancestry. We used summary statistics from the largest genome wide association studies (GWAS) to date in this population. We undertook multivariable MR to assess the non-glycemic effects of FI on HbA1c by adjusting for elevated fasting glucose (FG) and type 2 diabetes (T2D). We also explored the association between the triglyceride-glucose index (21), a surrogate measure of IR and HI and glycation gap (difference between measured HbA1c and predicted HbA1c from fasted glucose measurement), in a cohort of Canadian adults with normoglycemia and preT2D.

## Methods

### Cohorts

Demographic details of the cohorts used for MR analyses have been included in **Table 1** (22–28). GWAS summary statistics for FI, HbA1c and FG were derived from GWAS undertaken by MAGIC (22). Summary statistics for T2D were derived from DIAGRAM/GERA/UK Biobank consortia (26). All other summary statistics were from the UK Biobank (24, 25, 27).

**Table 1:** Cohort details for Mendelian Randomization (MR) analyses.

Trait	Population cohort	Mean Age	% Female	Sample size	Cases	Controls	PMID
Fasting Insulin (FI)	MAGIC	50.7	51.2	151,013	Not applicable	Not applicable	34059833
Type 2 Diabetes (T2D)	DIAGRAM/GERA/UK Biobank	54.1/63.3/56.9*	50.1/59.0/54.2*	655,666	61,714	593,952	30054458
Fasting Glucose (FG)	MAGIC	50.9	47.7	133,010	Not applicable	Not applicable	22885924
Hemoglobin (Hb)	UK Biobank	56.7	54.9	563,946	Not applicable	Not applicable	32888493
Red Cell Count (RCC)	UK Biobank	56.7	54.9	545,203	Not applicable	Not applicable	32888493
Reticulocytes (RETIC) ***	UK Biobank	56.7	54.9	408,112	Not applicable	Not applicable	32888494
HbA1c**	MAGIC	52.3	57.9	146,806	Not applicable	Not applicable	34059833
HbA1c**/***	UK Biobank	56.7	54.9	389,889	Not applicable	Not applicable	34017140

*Study-specific characteristics were not available for all UK Biobank data and was extrapolated from data available.

a Output from MRC IEU GWAS pipeline analysis using Phesant derived variables from UK Biobank, version 2: https://doi.org/10.5523/bris.pnoat8cxo0u52p6ynfaekeigi.

**To minimize overlap, bidirectional MR analyses with FI was undertaken with HbA1c measure in the UK Biobank, but for WHR adjusted for BMI analyses HbA1c was assessed in MAGIC.

***Estimated from available UK Biobank data (PMID 32888493) as data not available.

### Overlap between exposure and outcome cohorts

There is no reported overlap between the cohorts.

### Primary MR analyses

For our primary analysis, we undertook bidirectional inverse variance weighted (IVW) MR with FI as exposure (**Supplementary File 2**) and Hb, red cell count (RCC), reticulocyte count (RETIC) as outcomes. A p value of <0.05 was considered significant for primary and secondary analyses. We followed the recently published STROBE-MR reporting guidelines (Checklist in **Supplementary File 1**) (29). As we used publicly available summary statistics from GWAS, we did not seek institutional approval. Informed consent was obtained from the investigators from each participant in the original study.

### Secondary analyses

As we found a potential causal association between FI and erythrocytosis, we undertook univariable MR to investigate the association between FI and HbA1c followed by multivariable MR adjusted for FG, T2D or Hb. Adjustment for FG and T2D was not undertaken in combination due to concerns about collinearity (30). Adjustment for T2D and Hb in combination was not undertaken as the F-statistic was <10, indicative of a weak instrument.

MR assumptions: MR is based on three assumptions. First, the instrument is robustly associated with the exposure. Therefore, we only used SNPs that were genome-wide significantly associated for all the instruments (20). Second, that the instrument does not influence the outcome *via* another pathway other than the outcome i.e., no horizontal pleiotropy (20). Finally, the instrument is not influenced by any confounders (20). For univariable MR, we used inverse weighted MR (IVWMR) and additional sensitivity analyses including MR-Egger, weighted median, weighted mode and leave-one-out analyses.

IVWMR was performed by undertaking meta-analysis of the individual Wald ratio for each SNP in the instrument. By permitting a non-zero intercept, MR-Egger relaxes the assumption of no horizontal pleiotropy and returns an unbiased causal estimate, in the case of horizontal pleiotropy, providing that the horizontal pleiotropic effects are not correlated with the SNP-exposure effects (InSIDE assumption) (20, 31). The median effect of all SNPs in the instrument was used for analysis using weighted median MR, which permits SNPs with a greater effect on the association to be evaluated by weighting the contribution of each SNP by the inverse variance of its association with the outcome: this is robust even if only 50% of the SNPs satisfy all three MR assumptions (32). Finally, SNPs were clustered into groups based on similarity of causal effects for weighted mode MR, with the cluster with the largest number of SNPs deriving the causal effect estimate (33). Cochrane’s Q test was used to assess heterogeneity, while leave-one-out analyses were conducted to assess if any MR estimate was biased by a single SNP potentially with horizontal pleiotropic effect (20) and the F statistic was calculated to assess the strength of the instrument exposure (20, 34, 35).

Univariable MR was conducted using the “TwoSampleMR” package in R (R studio^®^ v1.3.1073 and R^®^ v4.0.3). Linkage disequilibrium (LD) pruning was used to select a proxy (r^2^>0.8) if a SNP was not directly matched from the 1000 Genomes project (Version 0.5.6, Released 2021-03-35). The “ggplot2” and “metaphor” packages in R were used to create plots. We undertook inverse variance weighted multivariable MR (IVW Multivariable MR) to assess the effect of FI on HbA1c after adjustment for FG and T2D and Hb (36). Multivariable MR was conducted using both the “TwoSampleMR”, “Multivariable MR” and “RMultivariable MR” packages in R (R studio^®^ v1.3.1073 and R^®^ v4.0.3), where the latter two packages assessed heterogeneity *via* Cochrane’s Q test and strength of the instrument *via* F statistics (34, 36). Plots were generated using “plotobject”.

### Observational study

Demographic details for this study have been included in **Table 2**. We received institutional approval from University Health Network (UHN) research ethics board for the observational study. As we analyzed anonymized data, we did not obtain consent from individual patients. We undertook analyses in a cohort of patients without T2D (n=7600 of whom 1096, i.e., 14.4%, had pre-T2D), who attended one of UHN’s, an academic health center in Toronto, Canada, outpatient clinics between 2006 and 2022. We excluded patients who had attended diabetes clinics in the prior 2 years, those with fasting glucose ≥ 7mmol/L, HbA1c ≥ 6.5%, age >65 years or <18 years or Hb outside the sex-specific normal range. We did not undertake analyses in patients with diabetes as we did not have access to their medical records and could not ascertain the type of diabetes or their medications (e.g., insulin and sodium glucose co-transporter 1 inhibitors), which can impact both glycemia and erythrocytosis (19, 37)

**Table 2:** Baseline characteristics for participants of observational study.

Characteristic	Healthy Participants, N = 6,504*	Participants with Pre-T2D, N = 1,096**	p-value*^2^*
Sex			0.076
Female	3,147 (48%)	562 (51%)	
Male	3,357 (52%)	534 (49%)	
Age (years)	50 (41, 58)	56 (49, 61)	<0.001
HbA1c (%)	5.40 (5.20, 5.60)	6.10 (6.00, 6.20)	<0.001
Fasting Glucose (mmol/L)	5.10 (4.70, 5.50)	5.70 (5.20, 6.20)	<0.001
Triglyceride (mmol/L)	1.16 (0.85, 1.68)	1.33 (0.99, 1.92)	<0.001
HDL Cholesterol (mmol/L)	1.29 (1.07, 1.57)	1.19 (1.01, 1.43)	<0.001
Hemoglobin (g/L)	145 (136, 154)	144 (134, 153)	<0.001
Triglyceride Glucose Index	8.46 (8.12, 8.85)	8.71 (8.36, 9.08)	<0.001
Predicted A1c, adjusted for age, sex (%)	5.04 (4.97, 5.11)	5.14 (5.06, 5.23)	<0.001
Glycation Gap***	0.36 (0.12, 0.57)	1.01 (0.89, 1.13)	<0.001

*Median (IQR); n (%).

**Wilcoxon rank sum test; Pearson’s Chi-squared test.

***Actual HbA1c – Predicted HbA1c.

Using R studio^®^ v1.3.1073 and R^®^ v4.0.3, predicted HbA1c was assessed based on regression analysis of fasting glucose adjusted for age and sex. To estimate the potential non-glycemic contribution to HbA1c, we assessed the glycation gap which is defined as the difference between measured HbA1c and predicted HbA1c (based on fasting glucose). We then assessed the association between triglyceride-glucose index (21), a surrogate measure of HI and IR, and the glycation gap. Triglyceride-glucose index (21) was calculated as ln [fasting triglyceride (mg/dL) × fasting plasma glucose (mg/dL)/2]. Correction factors of 88.57 and 18 were used to convert triglycerides to mg/L and fasting glucose to mg/dL, respectively.

The rms and lattice packages were used to fit a regression model and for estimation. The beta coefficient, standard error, y-intercept and p-value were analyzed in order to determine if there was an association between triglyceride-glucose index and glycation gap for all participants, those with pre-T2D (HbA1c 6-6.4% and fasting glucose 6-6.9 mmol/l) and those with normoglycemia (specified as HbA1c < 6% and fasting glucose <6 mmol/l). A p-value of < 0.05 was specified as being significant. The R^2^ and adjusted R^2^ were also analyzed to determine the fit of the model. An ANOVA table further analyzed if there was a linear relationship present.

Several diagnostic plots were created to test the presence of linearity and evaluate the fit of our model. A Normal Q-Q plot was created to test if the data had a normal distribution. If the data points fell onto a reasonably straight line, this would indicate a well fit model. Using the xyplot function, two plots were then created, both examining the residuals (residuals versus fitted values and residuals versus triglyceride-glucose index). If the plots produced a straight line, this would indicate a linear relationship. If the line was curved, this would indicate nonlinearity and splines would be required to analyze the cubic model.

Finally, the model was assessed for overfitting *via* validation. A set of random numbers was generated and the 0.632 Bootstrap method was used (38). The 0.632 Bootstrap method was chosen as to reduce bias by using correction factors. The R^2^ and mean squared error (MSE) were analyzed. An overfit model would produce a significantly different R^2^ and a higher MSE. Further, an optimism greater than 0.1 would suggest overfitting as well as a slope with shrinkage. A decrease in g-index would also be suggestive of overfitting. It should be noted that MSE and g-index may be difficult to interpret as they vary based on sample size and range of data. The same analyses were undertaken for triglyceride-glucose index on Hb.

## Results

### Primary analyses

#### Univariable MR analyses of FI and erythrocytosis (Hb, RCC, RETIC)

**Figure 1:**
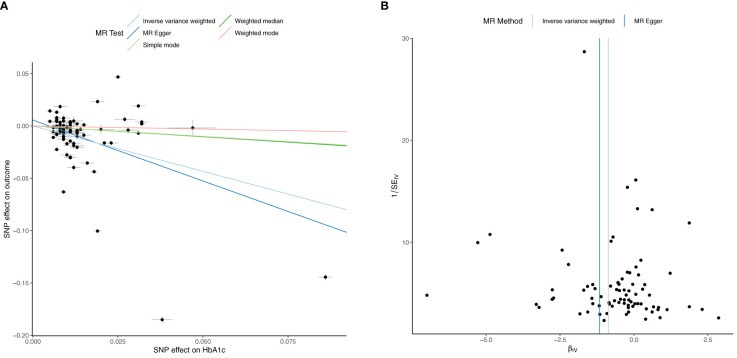
Univariable Mendelian Randomization (MR) Analysis — Exposure: fasting insulin (FI), Outcome: hemoglobin (Hb)— **(A)** Scatter plot showing the single nucleotide polymorphisms (SNPs) associated with FI against SNPs associated with Hb (vertical and horizontal black lines around points show 95% confidence intervals (CI) for five different Mendelian Randomization (MR) association tests **(B)** Funnel plot of the effect size against the inverse of the standard error for FI against Hb.

**Figure 2:**
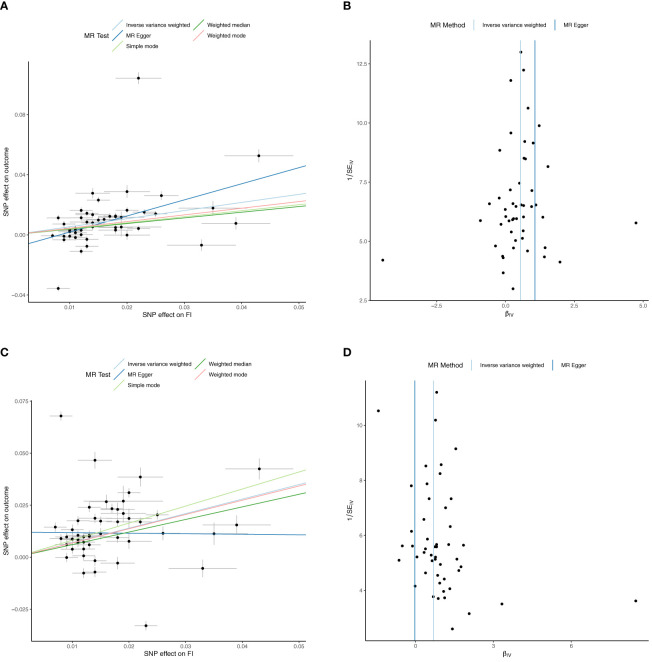
Univariable Mendelian Randomization (MR) Analysis — Exposure: fasting insulin (FI), Outcome: red cell count (RCC) and reticulocyte count (RETIC)— **(A)** Scatter plot showing the single nucleotide polymorphisms (SNPs) associated with FI against SNPs associated with RCC (vertical and horizontal black lines around points show 95% confidence intervals (CI) for five different Mendelian Randomization (MR) association tests **(B)** Funnel plot of the effect size against the inverse of the standard error for each SNP for FI against RCC **(C)** Scatter plot showing the single nucleotide polymorphisms (SNPs) associated with FI against SNPs associated with RETIC (vertical and horizontal black lines around points show 95% confidence intervals (CI) for five different Mendelian Randomization (MR) association tests **(D)** Funnel plot of the effect size against the inverse of the standard error for each SNP for FI against RETIC.

Univariable inverse variance weighted MR suggests increased FI increases Hb (b=0.54 ± 0.09, p=2.7x10^-10^, RCC (b=0.54 ± 0.012, p=5.38x10^-6^) and RETIC (b=0.70 ± 0.15, p=2.18x10^-6^), with concordant results with MR-Egger, weighted median, weighted mode and simple mode MR analyses (**Table 3**; **Figures 1**, **2**; **Supplementary File 5**).

**Table 3:** Univariable MR analyses of fasting insulin (FI) as exposure and hemoglobin (Hb), red cell count (RCC) and reticulocyte count (RETIC) as outcomes.

Method	β	Standard Error	P	Egger-Intercept	p_Egger_	Cochrane’s Q	Q df	p_Q_	I^2^	F
Univariable MR Analysis — Exposure: FI (49 SNPs, single nucleotide polymorphisms), Outcome: Hb										
MR Egger	0.678	0.231	0.005	-0.002	0.534	857.625	47	1.95x10^-149^	94.520	19.221
Weighted median	0.506	0.047	6.22x10^-27^							19.221
Inverse variance weighted	0.544	0.086	2.70x10 ^-10^			864.803	48	2.80x10^-150^	94.450	19.221
Simple mode	0.624	0.090	9.43x10^-9^							19.221
Weighted mode	0.575	0.063	4.54x10^-12^							19.221
Univariable MR Analysis — Exposure: FI (49 SNPs), Outcome: RCC										
MR Egger	1.070	0.307	0.001	-0.009	0.068	1520.582	47	8.23x10^-288^	96.909	19.221
Weighted median	0.377	0.055	7.66x10^-12^							19.221
Inverse variance weighted	0.539	0.118	5.38x10^-6^			1633.210	48	0	97.061	19.221
Simple mode	0.401	0.134	0.004							19.221
Weighted mode	0.443	0.098	3.85x10^-5^							19.221
Univariable MR Analysis — Exposure: FI (49 SNPs), Outcome: RETIC										
MR Egger	-0.026	0.379	0.946	0.012	0.045	1744.044	47	0	97.305	19.221
Weighted median	0.605	0.061	6.49x10^-23^							19.221
Inverse variance weighted	0.698	0.147	2.18x10^-6^			1902.129	48	0	97.477	19.221
Simple mode	0.819	0.116	6.18x10^-9^							19.221
Weighted mode	0.684	0.076	7.48x10^-12^							19.221

Inverse variance weighted MR suggests increased Hb (b=0.03 ± 0.01, p=0.02), RCC (b=0.02 ± 0.01, p=0.04) and RETIC (b=0.03 ± 0.01, p=0.002) might modestly increase FI, but MR-Egger, weighted median, weighted mode and simple mode MR analyses did not find evidence for potential causal association (**Supplementary Files 3**, **5**).

### Secondary analyses

#### Univariable and multivariable MR analyses of FI as exposure (adjusted for FG, T2D and Hb) and HbA1c as outcome

**Figure 3:**
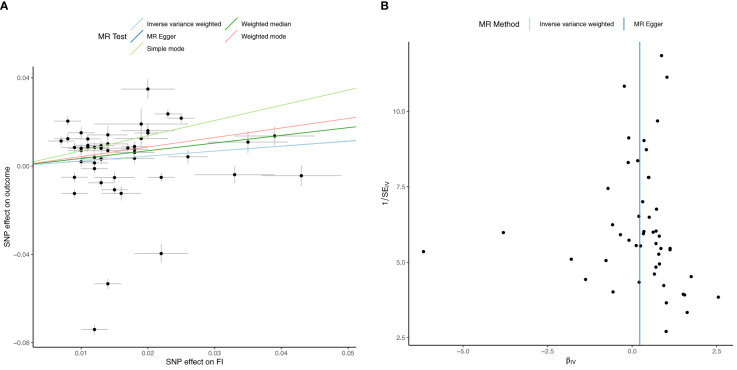
Univariable Mendelian Randomization (MR) Analysis — Exposure: fasting insulin (FI), Outcome: HbA1c— **(A)** Scatter plot showing the single nucleotide polymorphisms (SNPs) associated with FI against SNPs associated with HbA1c (vertical and horizontal black lines around points show 95% confidence intervals (CI) for five different Mendelian Randomization (MR) association tests **(B)** Funnel plot of the effect size against the inverse of the standard error for FI against HbA1c.

Univariable inverse variance weighted MR suggests that increased FI does not significantly increase HbA1c (b=0.23 ± 0.16, p=0.16) (**Table 4**; **Figure 3**; **Supplementary File 5**).

**Table 4:** Univariable MR analyses of fasting insulin (FI) and glycated hemoglobin (HbA1c) as outcome followed by multivariable MR adjusted for elevated fasting glucose (FG), type 2 diabetes (T2D) and increased hemoglobin (Hb).

Method	β	Standard Error	P	Egger-Intercept	p_Egger_	Cochrane’s Q	Q df	p_Q_	I^2^	F
Univariable MR Analysis — Exposure: FI (49 SNPs, single nucleotide polymorphisms), Outcome: HbA1c										
MR Egger	0.226	0.436	0.608	2.70x10^-5^	0.997	2610.360	47	0	98.199	19.221
Weighted median	0.348	0.055	1.72x10^-10^							19.221
Inverse variance weighted	0.227	0.162	0.162			2610.361	48	0	98.161	19.221
Simple mode	0.689	0.097	5.57x10^-9^							19.221
Weighted mode	0.432	0.118	6.42x10^-4^							19.221
Multivariable MR Analysis — Exposure: FI adjusted FG (30 SNPs), Outcome: HbA1c										
Inverse Variance Weighted	-0.221	0.133	0.096			1111.614	82	3.42x10^-180^	92.623	20.75
Multivariable MR Analysis — Exposure: FI adjusted T2D (21 SNPs), Outcome: HbA1c										
Inverse Variance Weighted	-0.305	0.126	0.016			1211.384	112	8.18x10^-184^	90.754	14.524
Multivariable MR Analysis — Exposure: FI adjusted Hb (16 SNPs), Outcome: HbA1c										
Inverse Variance Weighted	0.363	0.110	9.14x10^-4^			8336.312	403	0	95.166	4.547

Multivariable MR suggests FI decreases HbA1c after adjusting for T2D (b=-0.30 ± 0.13, p=0.02. After adjusting for Hb (b=0.36, p=9.14x10^-4^), FI increases HbA1c, but the F-statistic of <10 precludes definitive conclusion. There was no significant effect of FI on HbA1c after adjusting for FG alone (b=-0.221 ± 0.13, p=0.096) (**Table 4**; **Supplementary File 5**).

### Exploratory analyses

#### MR Analyses exploring association between Hb and HbA1c

MR suggests a bidirectional relationship between Hb and HbA1c. Univariable inverse variance weighted MR suggests increased Hb decreases HbA1c (b=-0.105, p=1.17x10^-13^) concordant with MR-Egger, weighted median and mode but not simple mode analyses (**Supplementary Files 4**, **5**). Reverse inverse variance weighted MR suggests increased HbA1c decreases Hb (b=-0.867, p=6.02x10^-7^) concordant with MR-Egger, weighted median and simple mode but not weighted mode analyses (**Supplementary Files 4**, **5**).

#### Observational study

**Figure 4:**
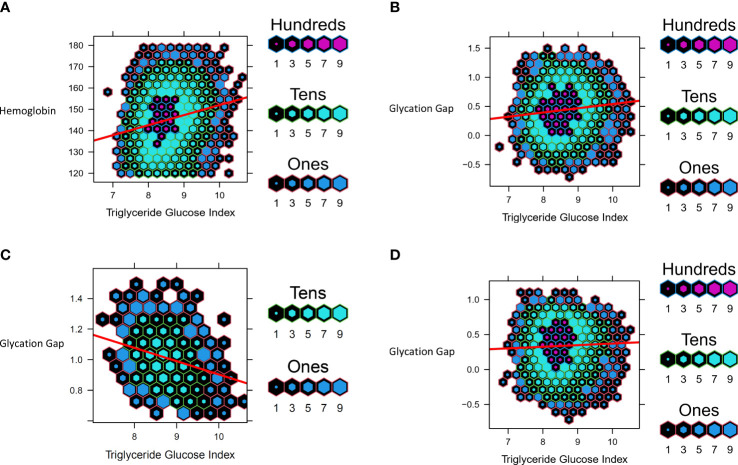
Data from UHN cohort. Hexbin plots represent number of study participants with the observed and calculated values, where the color and size of each individual hexagon correlates to the number of participants with the corresponding values. The red line represents the regression line for each cohort. **(A)** Association between Triglyceride Glucose Index and Hemoglobin **(B)** Association between Triglyceride Glucose Index and Glycation Gap in all participants **(C)** Association between Triglyceride Glucose Index and Glycation Gap in with pre-T2D **(D)** Association between Triglyceride Glucose Index and Glycation Gap in those with normoglycemia.

Cohort details and descriptive statistics regarding the 7600 participants can be found in **Tables 2**, **5**. Consistent with the MR analyses, increased TGI was associated with increased Hb (b=1.88 ± 0.19, p<0.001) (**Figure 4A**). Linear regression analysis yielded this equation for predicted HbA1c derived from fasting glucose and adjusted for age and sex: predicted HbA1c=4.163+0.172*(fasting glucose). In the cohort overall (the majority of whom had normoglycemia), (b=0.073 ± 0.008, p<0.0001) and in people with normoglycemia (b=0.023 ± 0.007, p<0.0001), increased triglyceride-glucose index was associated with an increase in glycation gap. However, among people with pre-T2D, increased triglyceride-glucose index was associated with a decreased glycation gap i.e., measured HbA1c was lower than that predicted by fasting glucose (b=-0.087 ± 0.009, p<0.0001) (**Figures 4B–D**).

**Table 5:** Linear regression for Triglyceride Glucose Index (exposure) on glycation gap* (outcome).

Population (n)	Beta	Standard error	y-intercept	p-value
Pre-T2D (1096)	-0.087	0.009	1.77	<0.0001
Healthy (6504)	0.023	0.007	0.14	<0.0001
HbA1c < 5% (681)	0.071	0.01	0.41	<0.0001
HbA1c 5 – 5.4% (2948)	0.047	0.005	0.6	<0.0001
HbA1c 5.5 – 5.9% (2875)	0.054	0.006	1.06	<0.0001

*Calculated as actual HbA1c – predicted HbA1c using model adjusted for age, sex (21).

For all analyses, the R^2^ adjusted and unadjusted were almost identical and the regression p-values were <0.005. Normal Q-Q plots for all data were suggestive of a normal distribution and good fit. Plots for residuals versus triglyceride-glucose index and residuals versus fitted values suggested linearity and that likely all relationships were accounted for in the model. Using the 0.632 Bootstrap method, validation was carried out and resulted in corrected R^2^ and corrected slopes that were relatively similar to the original values. The R optimism was found to be 0.0116, which was less than the cut-off of 0.1. The MSE increased by 1.57% and the g-index decreased by 3.97%. These results indicate that the model was not overfit.

## Discussion

Epidemiological data suggests that IR and HI are associated with increased erythrocytosis (16–18), which may plausibly be secondary to HI mediated erythrocytosis (19). Our MR analyses suggests that a causal association between HI and increased erythrocytosis. MR further suggests that increased FI after adjustment for T2D reduces HbA1c. MR also indicates a bidirectional inverse relationship between Hb and HbA1c. Collectively, this data suggest that HI mediated erythrocytosis might potentially lower HbA1c by non-glycemic effects with the transition from normoglycemia to T2D. Our observational data was concordant with the MR analyses. It showed that increased triglyceride-glucose index was associated with higher Hb. Further, increased triglyceride-glucose index associated with lower-than-expected HbA1c based on fasting glycemia. These findings await confirmation and assessment of clinical significance in well-designed prospective studies across the glycemic spectrum from normoglycemia to preT2D and T2D.

Increased FI is a recognized compensatory feature of IR. Some features of IR and HI such as increased hepatic glucose production are likely a consequence of reduced insulin action, while others such as hepatic steatosis and dyslipidemia are likely due to increased insulin action *via* signaling pathways that are not perturbed in IR (4, 5). *In vitro* studies suggest that insulin can increase erythrocytosis (19). This suggests that increased insulin action i.e., HI likely underpins the increased erythrocytosis seen with IR and HI. Further studies are needed to confirm these findings and explore underlying mechanisms and signaling pathways.

The potential non-glycemic impacts of increased fasting insulin on HbA1c might lead to lower-than-expected HbA1c based on glycemia and thus have implications for people with IR and HI during screening for preT2D and T2D. HbA1c is increasingly used to diagnose these conditions, in lieu of fasting glucose/oral glucose tolerance test measures, and to set glycemic targets for treatment (1, 9–12). Interestingly, observational data indicates that ~40% of people with T2D diagnosed based on more than one measure of elevated fasting glucose and/or post OGTT glucose, have HbA1c below the diabetes threshold (39). The potential non-glycemic effects of increased FI on HbA1c may also be particularly pertinent for weight loss induced T2D diabetes remission. HbA1c is the recommended glycemic parameter to define remission in a patient population with high prevalence of IR and HI (11).

The strengths of this study include MR analyses with the largest sample sizes in populations of European ancestry and likely minimal/no overlap between participants in the exposure and outcome cohorts. Our study has several limitations. The findings may not apply to other ethnic groups given that we used populations with European ancestry only. This may especially be a concern in populations with higher prevalence of hemoglobinopathies and red cell disorders (13, 40–43). Additionally, analyses were not stratified by sex which is a major determinant of body composition, IR and HI (44). For our observational data we did not have access to individual level data including medications and comorbidities. Due to these limitations, we also excluded patients with biochemical evidence of T2D as we could not reliably ascertain the type of diabetes and account for the potential impact of medications which might impact red cell parameters. We derived predicted HbA1c from fasting glucose and did not account for post-prandial readings which is a major limitation. Finally, we did not have measures of FI in the observational cohort and therefore used surrogate measures of IR and HI in our analyses.

In conclusion, our data suggests that increased FI, a feature of IR, may increase erythrocytosis and might potentially lower HbA1c independent of glycemia. As these findings might have implications for the diagnoses and management of preT2D and T2D, it merits well designed prospective confirmatory studies across the glycemic spectrum to confirm these findings and assess whether these effects are clinically relevant.

## Data availability statement

The original contributions presented in the study are included in the article/**Supplementary Material**. Further inquiries can be directed to the corresponding author.

## Ethics statement

The studies involving human participants were reviewed and approved by University Health Network. Written informed consent for participation was not required for this study in accordance with the national legislation and the institutional requirements.

## Author contributions

SD, AP, AN, and RK designed the study. All authors analyzed the data. AN, RK, and SD wrote the manuscript and all authors read and edited the manuscript. SD is the guarantor of this work. All authors contributed to the article and approved the submitted version.
