# Supplementary material for: Investigating the association between fasting insulin, erythrocytosis and HbA1c through Mendelian randomization and observational analyses
Source: Front Endocrinol (Lausanne). 2023 Mar 17;14:1146099. doi: 10.3389/fendo.2023.1146099 (PMC10064082; doi:10.3389/fendo.2023.1146099)
Supplement: Supplementary file 1 [file DataSheet_1.pdf]

Table of Contents — Supplemental Files

Supplementary File 1 – Cohorts for MR Analyses — Pg 2

Supplemental File 2 – STROBE Mendelian Randomization (MR) Checklist — Pg 3

Supplemental File 3 — Fasting Insulin instrument — Pg 11

Supplemental File 4– Reverse MR analyses. Exposure: Hb, RCC and RETIC. Outcome: Fasting insulin — Pg 14

Supplemental File 5 – Bidirectional MR analyses assessing the effect of Hb on HbA1c — Pg 15

Supplemental File 6 — Baseline characteristics for participants of observational study and descriptive statistics — Pg 22

Supplementary File 7 – Individual SNP Data and Leave-One-Out-Data Analyses exported from all univariable MR analyses conducted including primary analyses, secondary analyses and exploratory analyse —Pg 23

Supplementary File 1 – Cohorts

Summary statistics from the largest published genome wide association study (GWAS) in people of European ancestry were used in MR analyses (Supplementary Table 1) (18–24). Informed consent and institutional approval were previously obtained by the individual cohort investigators.

Table: Cohort details (all participants were of European descent)

Trait	Population cohort	Mean Age	% female	Sample size	Cases	Controls	PMID
Fasting Insulin (FI)	MAGIC	50.7	51.2	151,013	N/A	N/A	34059833
Waist-to-hip ratio (WHR)	GIANT/UK Biobank	55.5/56.9*	54.0/54.2*	694,649	N/A	N/A	30239722
Type 2 Diabetes (T2D)	DIAGRAM/ GERA/UK Biobank	54.1/63.3/ 56.9*	50.1/59.0/54.2*	655,666	61,714	593,952	30054458
Fasting Glucose (FG)	MAGIC	50.9	47.7	133,010	N/A	N/A	22885924
Haemoglobin (Hb)	UK Biobank	56.7	54.9	563,946	N/A	N/A	32888493
Red Cell Count (RCC)	UK Biobank	56.7	54.9	545,203	N/A	N/A	32888493
Reticulocytes (RETIC) ***	UK Biobank	56.7	54.9	408,112	N/A	N/A	32888494
HbA1c**	MAGIC	52.3	57.9	146,806	N/A	N/A	34059833
HbA1c**/***	UK Biobank	56.7	54.9	389,889	N/A	N/A	34017140

**Study-specific characteristics were not available for all UK Biobank data and was extrapolated from data available.*
[a]Output from MRC IEU GWAS pipeline analysis using Phesant derived variables from UK Biobank, version 2: https://doi.org/10.5523/bris.pnoat8cxo0u52p6ynfaekeigi
*** To minimize overlap, bidirectional MR analyses with FI was undertaken with HbA1c measure in the UK Biobank, but for WHR adjusted for BMI analyses HbA1c was assessed in MAGIC.*
**** Estimated from available UK Biobank data (PMID 32888493) as data not available*

Supplementary File 2. STROBE-MR checklist of recommended items to address in reports of Mendelian randomization studies[1 2]

Item No.	Section	Checklist item	Page No.	Relevant text from manuscript
1	**TITLE and ABSTRACT**	Indicate Mendelian randomization (MR) as the study's design in the title and/or the abstract if that is a main purpose of the study	1	A Mendelian randomization study investigating the potential causal association between fasting insulin and erythrocytosis and its non-glycemic impact on HbA1c
	**INTRODUCTION**			
2	**Background**	Explain the scientific background and rationale for the reported study. What is the exposure? Is a potential causal relationship between exposure and outcome plausible? Justify why MR is a helpful method to address the study question	4	Increasingly, haemoglobin A1c (HbA1c) has replaced fasting glucose and/or the 75 g oral glucose tolerance test to diagnose preT2D, T2D and T2D remission. HbA1c is also used to set glycemic targets for people with diabetes (7–9). Advantages to using HbA1c compared to fasting glucose, include convenience and use of an assay that is standardized, stable, reproducible with limited intraindividual variability (1,10). It provides an average measure of glycemia in the preceding 2 to 3 months (1). However, altered red cell lifespan and erythrocytosis, which is not routinely assessed, can affect HbA1c measurement by non-glycemic pathways, which has implications in patients with red cell disorders and haemoglobinopathies (1,11). In people without T2D, including those with preT2D, non-glycemic parameters are a major predictor of HbA1c: higher haemoglobin associates with lower HbA1c (12,13). Observational studies have also shown an association between IR/HI and increased haemoglobin and red cell count (14–16), but whether this association is causal is not established, nor is it known whether this impacts HbA1c measurement by non-glycemic pathways.
3	**Objectives**	State specific objectives clearly, including pre-specified causal hypotheses (if any). State that MR is a method that, under specific assumptions, intends to estimate causal effects	5	We undertook bidirectional MR to investigate potential causal associations between fasting insulin (FI) and erythrocytosis (haemoglobin, red cell count and reticulocyte count: primary outcome) in people of European ancestry using summary statistics from the largest genome wide association studies (GWAS). For our secondary outcome, we undertook multivariable MR to assess the non-glycemic effects of FI on HbA1c after adjusting for

					elevated fasting glucose (FG) and type 2 diabetes (T2D).
	**METHODS**				
4	**Study design and data sources**		Present key elements of the study design early in the article. Consider including a table listing sources of data for all phases of the study. For each data source contributing to the analysis, describe the following:		Summary statistics from the largest published genome wide association study (GWAS) in people of European ancestry were used in MR analyses
		a)	Setting: Describe the study design and the underlying population, if possible. Describe the setting, locations, and relevant dates, including periods of recruitment, exposure, follow-up, and data collection, when available.	5-6	Summary statistics from the largest published genome wide association study (GWAS) in people of European ancestry were used in MR analyses<br><br>Table 1 – cohort details
		b)	Participants: Give the eligibility criteria, and the sources and methods of selection of participants. Report the sample size, and whether any power or sample size calculations were carried out prior to the main analysis	18	Table 1 – cohort details
		c)	Describe measurement, quality control and selection of genetic variants	5-6	Table 1 contains specific populations used. Summary statistics were taken from published GWAS
		d)	For each exposure, outcome, and other relevant variables, describe methods of assessment and diagnostic criteria for diseases	6	See above for b) c) d)
		e)	Provide details of ethics committee approval and participant informed consent, if relevant		Informed consent and institutional approval were previously obtained by the individual cohort investigators.
5	**Assumptions**		Explicitly state the three core IV assumptions for the main analysis (relevance, independence and exclusion restriction) as well assumptions for any additional or sensitivity analysis	6	MR assumptions: MR is based on three assumptions. First, the instrument is robustly associated with the exposure, therefore we only used SNPs that were genome-wide significantly associated for all the instruments (17). Second that the instrument does not influence the outcome via another pathway other than the outcome i.e. no horizontal pleiotropy (17). Finally, the instrument is not influenced by any confounders (17).
6	**Statistical methods: main analysis**		Describe statistical methods and statistics used		

		a)	Describe how quantitative variables were handled in the analyses (i.e., scale, units, model)	7-9	Betas are provided for continuous variables.
		b)	Describe how genetic variants were handled in the analyses and, if applicable, how their weights were selected	6	Genetic variants were weighted based on effect size in prior meta-GWAS.
		c)	Describe the MR estimator (e.g. two-stage least squares, Wald ratio) and related statistics. Detail the included covariates and, in case of two-sample MR, whether the same covariate set was used for adjustment in the two samples	6	IVWMR was performed by undertaking meta-analysis of the individual Wald ratio for each SNP in the instrument. By permitting a non-zero intercept, MR-Egger relaxes the assumption of no horizontal pleiotropy and returns an unbiased causal estimate, in the case of horizontal pleiotropy, providing that the horizontal pleiotropic effects are not correlated with the SNP-exposure effects (InSIDE assumption) (17,27).
		d)	Explain how missing data were addressed	7	Linkage disequilibrium (LD) pruning was used to select a proxy (r2>0.8) if a SNP was not directly matched from the 1000 Genomes project
		e)	If applicable, indicate how multiple testing was addressed	NA	NA
7	**Assessment of assumptions**		Describe any methods or prior knowledge used to assess the assumptions or justify their validity	4	In people without T2D, including those with preT2D, non-glycemic parameters are a major predictor of HbA1c: higher haemoglobin associates with lower HbA1c (12,13). Observational studies have also shown an association between IR/HI and increased haemoglobin and red cell count (14–16), but whether this association is causal is not established, nor is it known whether this impacts HbA1c measurement by non-glycemic pathways.
8	**Sensitivity analyses and additional analyses**		Describe any sensitivity analyses or additional analyses performed (e.g. comparison of effect estimates from different approaches, independent replication, bias analytic techniques, validation of instruments, simulations)	6-7	For univariable MR, we used inverse weighted MR (IVWMR) and additional sensitivity analyses including MR-Egger, weighted median, weighted mode and leave-one-out analyses.<br>Cochrane's Q test was used to assess heterogeneity, while leave-one-out analyses were conducted to assess if any MR estimate was biased by a single SNP potentially with horizontal pleiotropic effect (17) and the F statistic was calculated to assess the strength of the instrument exposure (17,30,31).
9	**Software and pre-registration**				

		a)	Name statistical software and package(s), including version and settings used	7	Univariable MR was conducted using the “TwoSampleMR” package in R (R studio® v1.3.1073 and R® v4.0.3). Linkage disequilibrium (LD) pruning was used to select a proxy (r2>0.8) if a SNP was not directly matched from the 1000 Genomes project (Version 0.5.6, Released 2021-03-35). The “ggplot2” and “metaphor” packages in R were used to create plots. We undertook inverse variance weighted multivariable MR (IVW Multivariable MR) to assess the effect of FI on HbA1c after adjustment for FG and T2D as well as Hb (32). Multivariable MR was conducted using both the “TwoSampleMR”, “Multivariable MR” and “RMultivariable MR” packages in R (R studio® v1.3.1073 and R® v4.0.3) where the latter two packages assessed heterogeneity via Cochrane’s Q test and strength of the instrument via F statistics (30,32). Plots were; generated using “plotobject”.
		b)	State whether the study protocol and details were pre-registered (as well as when and where)		The study was not pre-registered.
	**RESULTS**				
10	**Descriptive data**				
		a)	Report the numbers of individuals at each stage of included studies and reasons for exclusion. Consider use of a flow diagram	18	Table 1 – cohort details
		b)	Report summary statistics for phenotypic exposure(s), outcome(s), and other relevant variables (e.g. means, SDs, proportions)	18	Table 1 – cohort details
		c)	If the data sources include meta-analyses of previous studies, provide the assessments of heterogeneity across these studies	18	Table 1 – cohort details – PMID to original GWAS studies are provided
		d)	For two-sample MR:<br>i. Provide justification of the similarity of the genetic variant-exposure associations between the exposure and outcome samples<br>ii. Provide information on the number of individuals who overlap between the exposure and outcome studies	18 and 7	Cohort details in Table 1: exposure and outcome samples have similar mean age and %female.<br>For our primary and secondary outcomes, there is no reported overlap between the cohorts. For our analyses of WHR adjusted for BMI and erythrocytosis, 456,426 participants from the UK Biobank composed approximately 67% of the GIANT/UK Biobank GWAS for WHR adjusted for BMI.
11	**Main results**				

		a)	Report the associations between genetic variant and exposure, and between genetic variant and outcome, preferably on an interpretable scale	8-9	Associations were reported as increases or decreases outcome.
		b)	Report MR estimates of the relationship between exposure and outcome, and the measures of uncertainty from the MR analysis, on an interpretable scale, such as odds ratio or relative risk per SD difference	8-9	Beta and standard error were provided with p-values for continuous variables.
		c)	If relevant, consider translating estimates of relative risk into absolute risk for a meaningful time period	NA	NA
		d)	Consider plots to visualize results (e.g. forest plot, scatterplot of associations between genetic variants and outcome versus between genetic variants and exposure)	8-9	Figures and Supplemental Files – There are figures of forest plots, scatterplots and other graphs displaying the results visually.
12	**Assessment of assumptions**				
		a)	Report the assessment of the validity of the assumptions	8-9	MR-Egger intercept with p-value was reported as measure of horizontal pleiotropy for all significant associations.
		b)	Report any additional statistics (e.g., assessments of heterogeneity across genetic variants, such as I^2, Q statistic or E-value)	8-9	Assessment of heterogeneity across genetic variants were provided for all analyses including I2 and Q statistics.
13	**Sensitivity analyses and additional analyses**				
		a)	Report any sensitivity analyses to assess the robustness of the main results to violations of the assumptions	8-9	MR- Egger, weighted-median and weighted-mode analyses were completed.
		b)	Report results from other sensitivity analyses or additional analyses	8-9	Visualization of scatter plots, funnel plots and leave-one-out analyses were completed.
		c)	Report any assessment of direction of causal relationship (e.g., bidirectional MR)	8-9	Bidirectional MR was completed for all univariable MR analyses.
		d)	When relevant, report and compare with estimates from non-MR analyses	NA	NA
		e)	Consider additional plots to visualize results (e.g., leave-one-out analyses)	8-9	See figures and supplemental files.
	**DISCUSSION**				
14	**Key results**		Summarize key results with reference to study objectives	10	Our MR analysis suggests that this association is causal and further suggests that increased FI after adjustment for FG and T2D may reduce HbA1c.

					MR also indicates a bidirectional inverse relationship between Hb and HbA1c. Collectively, these data suggest that increased IR/HI mediated erythrocytosis may potentially lower HbA1c by non-glycemic effects.
15	**Limitations**		Discuss limitations of the study, taking into account the validity of the IV assumptions, other sources of potential bias, and imprecision. Discuss both direction and magnitude of any potential bias and any efforts to address them	11	Our findings may not apply to other ethnic groups given that we used populations with European ancestry only. This may especially be a concern in populations with higher prevalence of hemoglobinopathies and red cell disorders (38–42). Additionally, analyses were not stratified by sex, which is a major determinant of body composition and IR/HI (34). There may also be a relationship between sex hormones such as estradiol and sex hormone binding globulin and HbA1c (43). Finally, there was significant overlap between our exposure and outcome cohorts for our exploratory analyses of the association between waist-hip ratio, erythrocytosis and HbA1c.
16	**Interpretation**				
		a)	Meaning: Give a cautious overall interpretation of results in the context of their limitations and in comparison with other studies	10	Our MR analysis suggests that this association is causal and further suggests that increased FI after adjustment for FG and T2D may reduce HbA1c. MR also indicates a bidirectional inverse relationship between Hb and HbA1c. Collectively, these data suggest that increased IR/HI mediated erythrocytosis may potentially lower HbA1c by non-glycemic effects. In exploratory analyses we also report that increased WHR adjusted for BMI, a phenotype associated with IR/HI (26,33) may increase erythrocytosis but we were unable to confirm a significant non-glycemic impact on HbA1c warranting caution in interpreting the data.
		b)	Mechanism: Discuss underlying biological mechanisms that could drive a potential causal relationship between the investigated exposure and the outcome, and whether the gene-environment equivalence assumption is reasonable. Use causal language carefully, clarifying that IV estimates may provide causal effects only under certain assumptions	10	Increased FI is a recognised manifestation of IR. Some features of IR/HI such as increased hepatic glucose production are likely a consequence of reduced insulin action, while others such as hepatic steatosis and dyslipidemia are likely due to increased insulin action via signaling pathways that are not perturbed in IR (35). In vitro studies suggest that insulin can increase erythrocytosis (36), suggesting that increased insulin action may underlie the increased in erythrocytosis with IR/HI.

				Further studies are needed to confirm these findings and explore underlying mechanisms.
		c) Clinical relevance: Discuss whether the results have clinical or public policy relevance, and to what extent they inform effect sizes of possible interventions	11	In conclusion, our data suggests that increased FI may increase erythrocytosis and potentially lower HbA1c by non-glycemic effects. These findings might have implications for the diagnoses of preT2D and T2D, its treatment and remission and merits further confirmatory studies.
17	**Generalizability**	Discuss the generalizability of the study results (a) to other populations, (b) across other exposure periods/timings, and (c) across other levels of exposure	11	In conclusion, our data suggests that increased FI may increase erythrocytosis and potentially lower HbA1c by non-glycemic effects. These findings might have implications for the diagnoses of preT2D and T2D, its treatment and remission and merits further confirmatory studies.<br><br>Our findings may not apply to other ethnic groups given that we used populations with European ancestry only. This may especially be a concern in populations with higher prevalence of hemoglobinopathies and red cell disorders (38–42). Additionally, analyses were not stratified by sex, which is a major determinant of body composition and IR/HI (34). There may also be a relationship between sex hormones such as estradiol and sex hormone binding globulin and HbA1c (43). Finally, there was significant overlap between our exposure and outcome cohorts for our exploratory analyses of the association between waist-hip ratio, erythrocytosis and HbA1c.
	**OTHER INFORMATION**			
18	**Funding**	Describe sources of funding and the role of funders in the present study and, if applicable, sources of funding for the databases and original study or studies on which the present study is based	11	SD is funded by CIHR, Heart & Stroke Foundation of Canada and Banting & Best Diabetes Centre (DH Gales Family Charitable Foundation New Investigator Award and a Reuben & Helene Dennis Scholar in Diabetes Research).
19	**Data and data sharing**	Provide the data used to perform all analyses or report where and how the data can be accessed, and reference these sources in the article. Provide the statistical code needed to reproduce the results in the article, or report whether the code is publicly accessible and if so, where		All data is public access. Please refer to Table 1 for cohort details.<br><br>TwoSampleMR R code and MVMR code is also publicly available.

20	**Conflicts of Interest**	All authors should declare all potential conflicts of interest	12	None

This checklist is copyrighted by the Equator Network under the Creative Commons Attribution 3.0 Unported (CC BY 3.0) license.

1. Skrivankova VW, Richmond RC, Woolf BAR, Yarmolinsky J, Davies NM, Swanson SA, et al. Strengthening the Reporting of Observational Studies in Epidemiology using Mendelian Randomization (STROBE-MR) Statement. JAMA. 2021;under review.

2. Skrivankova VW, Richmond RC, Woolf BAR, Davies NM, Swanson SA, VanderWeele TJ, et al. Strengthening the Reporting of Observational Studies in Epidemiology using Mendelian Randomisation (STROBE-MR): Explanation and Elaboration. BMJ. 2021;375:n2233.

Supplementary File 3. Fasting Insulin instrument.

Chromosome	Phenotype	SNP	effect_allele	other_allele	eaf	beta	se	pval
1	FI	rs6674544	A	G	0.57	0.018	0.002	6.97E-21
2	FI	rs77935490	A	T	0.21	0.014	0.003	1.07E-08
2	FI	rs61007968	C	G	0.21	0.011	0.003	2.63E-06
2	FI	rs1260326	T	C	0.39	-0.023	0.002	8.42E-38
2	FI	rs2252867	T	C	0.64	0.007	0.002	1.46E-05
2	FI	rs6713419	T	C	0.63	0.019	0.002	1.44E-24
2	FI	rs75265117	C	G	0.88	0.028	0.003	1.54E-24
2	FI	rs1128249	T	G	0.39	-0.02	0.002	7.71E-28
2	FI	rs13389219	T	C	0.39	-0.02	0.002	5.84E-28
2	FI	rs2943646	A	G	0.37	-0.025	0.002	8.47E-39
2	FI	rs2972145	T	C	0.37	-0.025	0.002	6.08E-38
2	FI	rs17508368	T	C	0.06	0.019	0.004	2.12E-06
3	FI	rs308971	A	G	0.87	-0.022	0.003	3.91E-13
3	FI	rs11712037	C	G	0.87	0.028	0.003	2.10E-21
3	FI	rs35000407	T	G	0.86	0.026	0.003	1.50E-21
3	FI	rs9819511	T	C	0.3	0.014	0.002	2.26E-08
3	FI	rs17331151	T	C	0.11	-0.016	0.003	1.52E-08
3	FI	rs11708067	A	G	0.78	-0.014	0.002	1.30E-09
3	FI	rs62271373	A	T	0.06	0.026	0.005	1.60E-08
4	FI	rs2276936	A	C	0.5	-0.012	0.002	4.25E-11
4	FI	rs3775380	A	G	0.5	-0.012	0.002	1.48E-11
4	FI	rs9884482	T	C	0.61	-0.013	0.002	2.88E-11
4	FI	rs10010325	A	C	0.48	0.012	0.002	4.05E-11
4	FI	rs7654571	A	G	0.24	0.012	0.002	7.77E-08
4	FI	rs11727676	T	C	0.91	-0.02	0.004	2.90E-08
4	FI	rs6855363	T	C	0.68	0.013	0.002	4.04E-08
5	FI	rs4865796	A	G	0.68	0.017	0.002	7.33E-17
5	FI	rs459193	A	G	0.27	-0.018	0.002	1.12E-18
5	FI	rs3936511	A	G	0.82	-0.019	0.003	2.81E-14
5	FI	rs1023667	A	G	0.27	-0.01	0.002	5.70E-07
6	FI	rs116684538	A	T	0.22	-0.014	0.003	1.31E-07
6	FI	rs116141873	T	G	0.04	0.043	0.006	1.42E-11
6	FI	rs2780215	A	G	0.96	0.039	0.006	1.06E-09

Supplementary File 3. Fasting Insulin instrument.

6	FI	rs1187129	A	G	0.03	-0.029	0.007	1.41E-05
6	FI	rs5875762	C	CA	0.31	-0.008	0.003	1.53E-03
6	FI	rs998584	A	C	0.49	0.012	0.002	2.31E-10
6	FI	rs9472135	T	C	0.7	0.011	0.002	4.21E-08
6	FI	rs1474696	A	G	0.49	-0.015	0.002	3.02E-16
6	FI	rs2745353	T	C	0.52	0.015	0.002	4.63E-16
6	FI	rs632057	T	G	0.4	0.008	0.002	1.37E-05
6	FI	rs73013411	A	C	0.13	-0.018	0.003	2.08E-08
6	FI	rs4709746	T	C	0.13	-0.018	0.003	2.97E-08
7	FI	rs2282930	A	G	0.23	-0.011	0.003	1.67E-05
7	FI	rs2108349	A	G	0.66	-0.012	0.002	1.13E-08
7	FI	rs848494	A	G	0.74	0.009	0.002	9.25E-06
7	FI	rs13234269	A	T	0.48	-0.011	0.002	1.27E-08
7	FI	rs972283	A	G	0.47	-0.011	0.002	1.09E-08
8	FI	rs330945	T	C	0.63	0.014	0.002	1.82E-11
8	FI	rs7012637	A	G	0.48	-0.022	0.002	1.48E-29
8	FI	rs7012814	A	G	0.48	-0.022	0.002	8.34E-30
8	FI	rs4841132	A	G	0.11	0.026	0.003	3.83E-20
8	FI	rs12541800	A	G	0.49	0.011	0.002	6.17E-07
8	FI	rs12056334	A	C	0.13	0.005	0.003	1.16E-01
8	FI	rs13258890	T	C	0.77	0.013	0.003	2.77E-08
9	FI	rs4339696	T	G	0.48	0.009	0.002	1.52E-07
9	FI	rs11138325	T	C	0.58	-0.012	0.002	5.39E-08
9	FI	rs75179845	T	C	0.91	-0.022	0.004	6.05E-11
9	FI	rs8176693	T	C	0.1	0.02	0.003	1.10E-10
10	FI	rs10761762	T	C	0.51	0.008	0.002	1.98E-05
10	FI	rs118164457	T	C	0.96	-0.035	0.006	3.86E-10
10	FI	rs12769346	T	G	0.86	-0.015	0.003	1.20E-08
10	FI	rs11191559	T	C	0.09	-0.014	0.003	1.27E-05
10	FI	rs7903146	T	C	0.27	-0.012	0.002	1.24E-09
10	FI	rs7071062	T	C	0.97	0.019	0.007	1.19E-02
11	FI	rs2845885	T	C	0.93	-0.02	0.004	1.18E-08

Supplementary File 3. Fasting Insulin instrument.

11	FI	rs3781926	T	C	0.36	0.009	0.002	3.26E-05
12	FI	rs12369443	A	G	0.78	0.01	0.002	6.66E-06
12	FI	rs2054435	A	G	0.22	-0.015	0.003	9.50E-09
12	FI	rs6487237	A	C	0.78	0.015	0.003	4.68E-09
12	FI	rs10842708	A	G	0.74	-0.01	0.002	1.08E-05
12	FI	rs111264094	C	G	0.97	0.057	0.009	1.64E-09
12	FI	rs1351394	T	C	0.49	-0.011	0.002	2.71E-09
12	FI	rs7968682	T	G	0.51	0.011	0.002	3.17E-09
12	FI	rs73343765	A	G	0.006	-0.19	0.053	2.14E-04
12	FI	rs1402013	A	G	0.35	-0.009	0.002	1.31E-06
12	FI	rs860598	A	G	0.82	0.018	0.003	6.88E-12
12	FI	rs35747	A	G	0.82	0.017	0.003	2.68E-11
12	FI	rs7133378	A	G	0.32	-0.013	0.002	6.00E-11
12	FI	rs7975482	A	G	0.67	0.012	0.002	1.97E-10
13	FI	rs9521730	A	G	0.34	-0.009	0.002	1.15E-06
16	FI	rs2024449	T	C	0.57	0.01	0.002	4.81E-07
18	FI	rs12454712	T	C	0.58	0.014	0.003	1.78E-09
19	FI	rs1799815	A	G	0.06	0.033	0.006	9.24E-08
19	FI	rs4804833	A	G	0.39	0.01	0.002	6.59E-06
19	FI	rs10422861	T	C	0.67	-0.013	0.002	6.65E-11
19	FI	rs731839	A	G	0.66	-0.012	0.002	3.87E-11
19	FI	rs339525	T	G	0.26	-0.007	0.003	2.07E-04
19	FI	rs200172871	C	CT	0.48	0.011	0.003	1.71E-05
20	FI	rs979012	T	C	0.34	0.011	0.002	4.81E-07
20	FI	rs285171	C	G	0.16	-0.012	0.003	8.94E-06
20	FI	rs1999536	C	G	0.55	-0.012	0.002	1.32E-09
20	FI	rs1206760	A	G	0.54	-0.011	0.002	8.82E-10
21	FI	rs200678953	T	TATATGTTATATAC	0.37	0.014	0.003	2.04E-08
X	FI	rs2497942	C	G	0.81	-0.011	0.003	1.61E-06
X	FI	rs12007422	T	G	0.62	0.011	0.002	1.10E-06

Supplemental File 4: **Reverse MR analyses. Exposure: Hb, RCC and RETIC. Outcome: Fasting insulin**

Method	B	Standard Error	P	Egger-Intercept	p_{Egger}	Cochran's Q	Q df	p_Q	I^2	F
**Univariable MR Analysis — Exposure: Hb (184 SNPs, single nucleotide polymorphisms), Outcome: FI**										
MR Egger	-0.008	0.026	0.746	0.001	0.055	805.243	182	$3.33x10^{-79}$	77.398	95.738
Weighted median	-0.021	0.013	0.098							95.738
Inverse variance weighted	0.033	0.014	0.017			821.763	183	$1.13x10^{-81}$	77.731	95.738
Simple mode	-0.032	0.029	0.266							95.738
Weighted mode	-0.020	0.012	0.102							95.738
**Univariable MR Analysis — Exposure: RCC (214 SNPs), Outcome: FI**										
MR Egger	-0.014	0.021	0.504	0.001	0.050	736.709	212	$3.93x10^{-59}$	71.223	147.934
Weighted median	0.000	0.010	1.000							147.934
Inverse variance weighted	0.022	0.011	0.043			750.172	213	$5.88x10^{-61}$	71.607	147.934
Simple mode	-0.025	0.018	0.168							147.934
Weighted mode	-0.001	0.011	0.938							147.934
**Univariable MR Analysis — Exposure: RETIC (210 SNPs), Outcome: FI**										
MR Egger	-0.001	0.017	0.931	0.001	0.024	761.249	208	$4.25x10^{-64}$	72.676	316.202
Weighted median	0.011	0.010	0.259							316.202
Inverse variance weighted	0.031	0.010	0.002			780.233	209	$7.81x10^{-67}$	73.213	316.202
Simple mode	0.008	0.021	0.694							316.202
Weighted mode	0.003	0.012	0.768							316.202

Supplementary File 5 — Bidirectional MR analyses assessing the effect of Hb on HbA1c

Description of Different Figure Types

A) Scatter plot showing the single nucleotide polymorphisms (SNPs) associated with Hb against SNPs associated with HbA1c as the outcome (vertical and horizontal black lines around points show 95% confidence intervals (CI)) for five different Mendelian Randomization (MR) association tests.

B) Funnel plot of the effect size against the inverse of the standard error for each SNP.

Exposure: Hb, Outcome: HbA1c
Figures of Scatter Plot of MR Methods (A) and Funnel Plot (B)

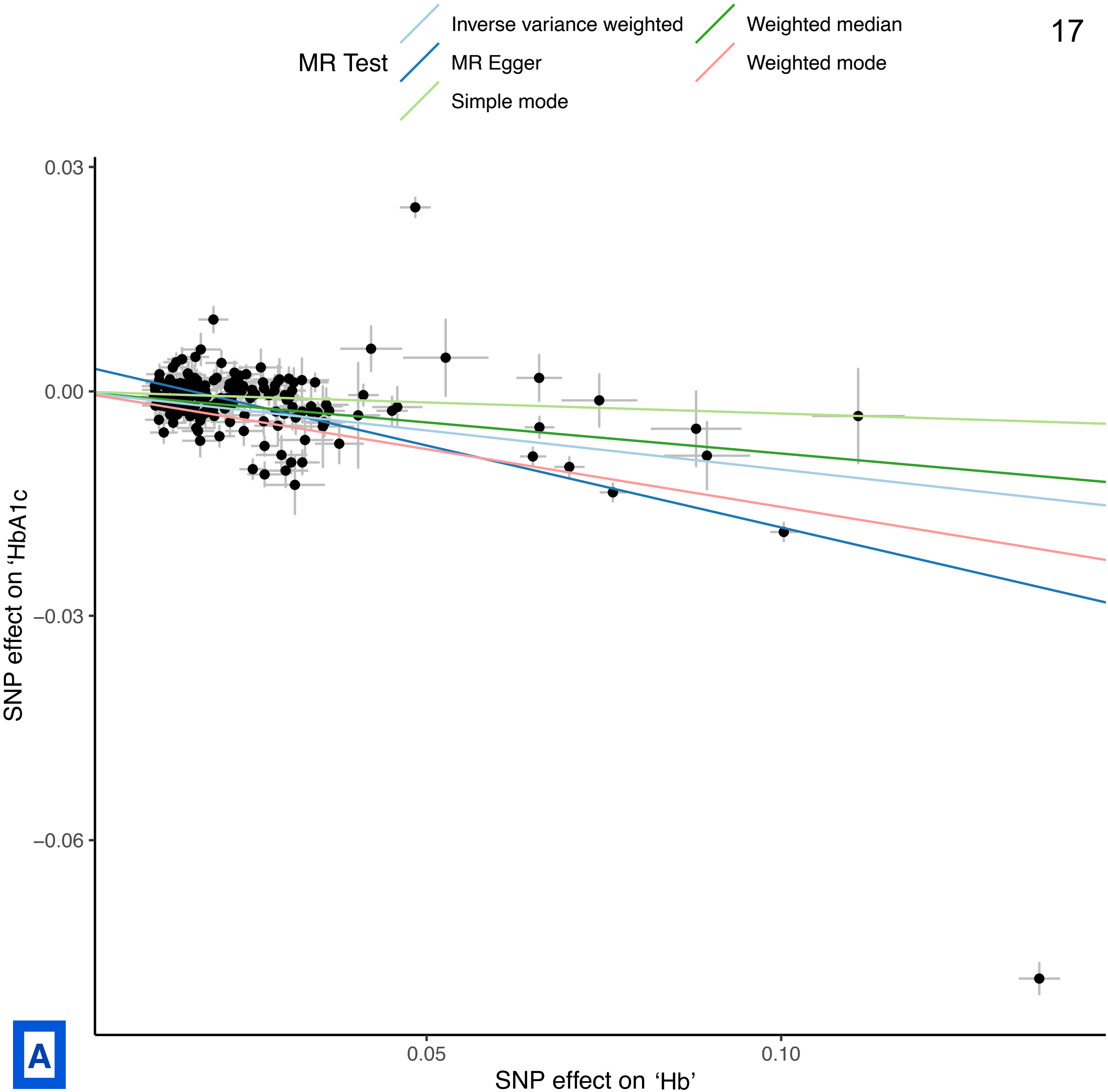
MR Test
Inverse variance weighted
MR Egger
Simple mode
Weighted median
Weighted mode
0.03
0.00
−0.03
−0.06
SNP effect on ‘HbA1c
0.05
0.10
SNP effect on ‘Hb’
A

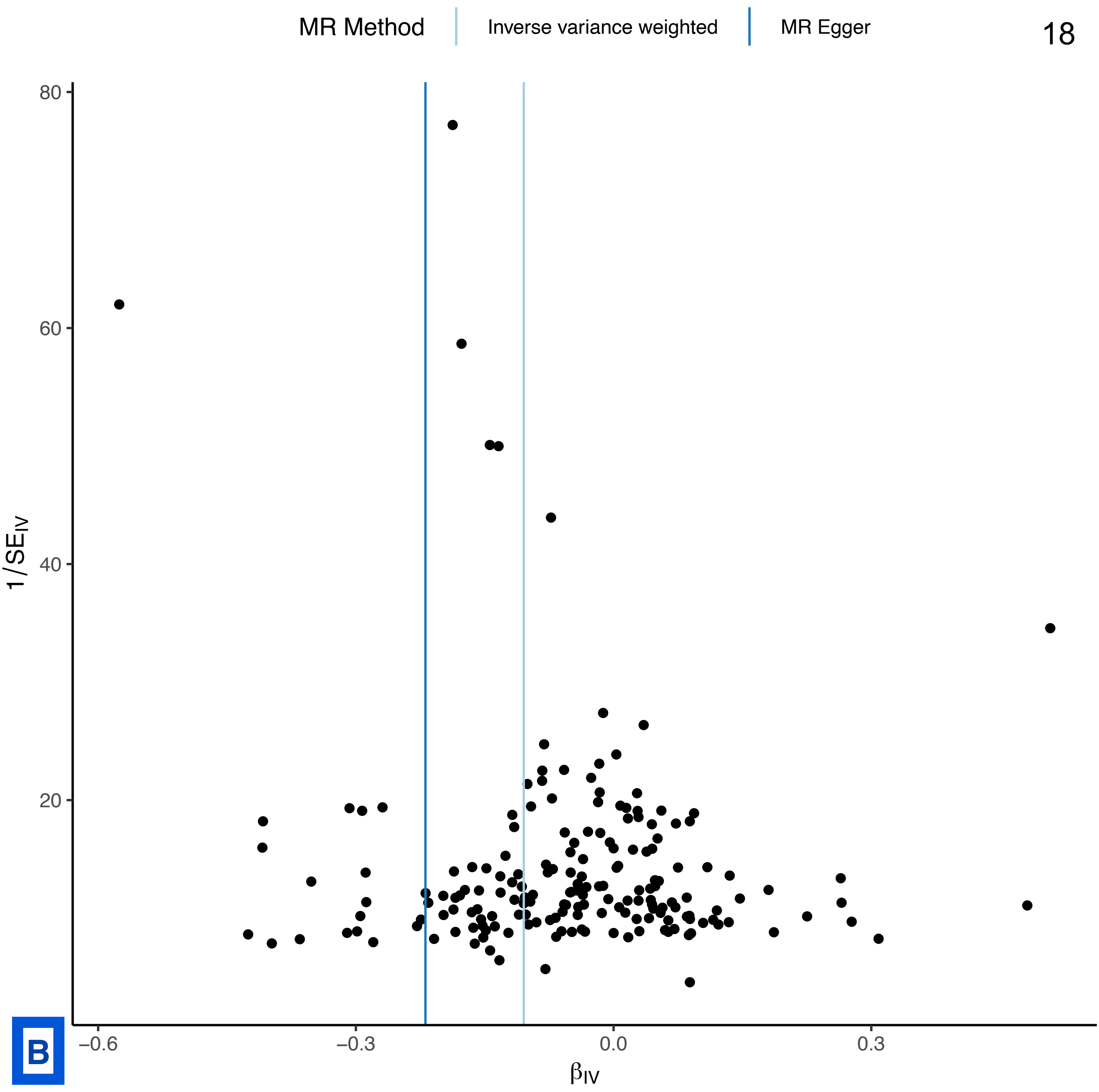
MR Method
Inverse variance weighted
MR Egger
80
60
40
20
1/SE$_{IV}$
−0.6
−0.3
0.0
0.3
β_{IV}
B

Exposure: HbA1c, Outcome: Hb
Figures of Scatter Plot of MR Methods (A) and Funnel Plot (B)

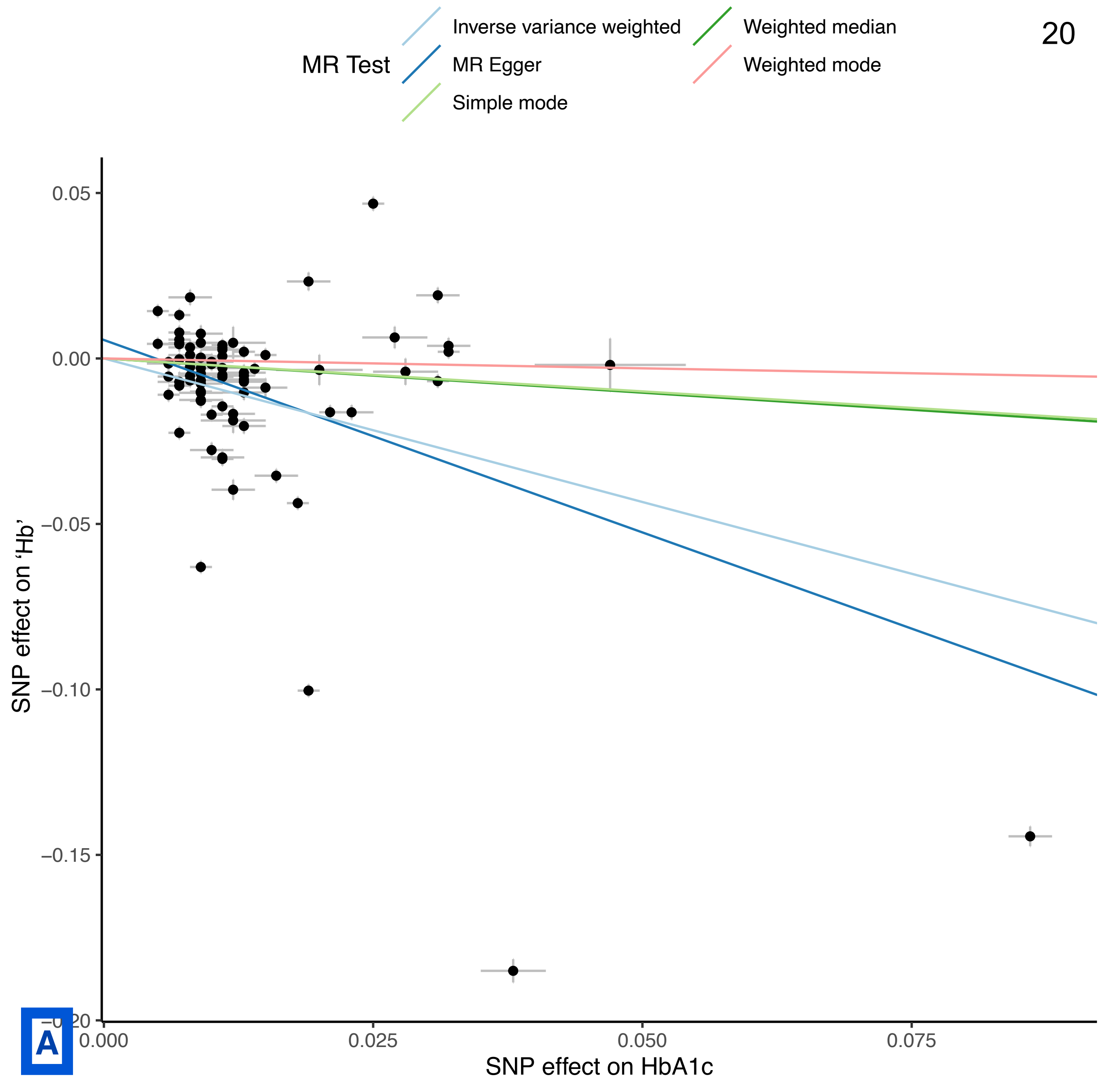
MR Test
Inverse variance weighted
MR Egger
Simple mode
Weighted median
Weighted mode
0.05
0.00
−0.05
−0.10
−0.15
−0.20
SNP effect on 'Hb'
0.000
0.025
0.050
0.075
SNP effect on HbA1c
A

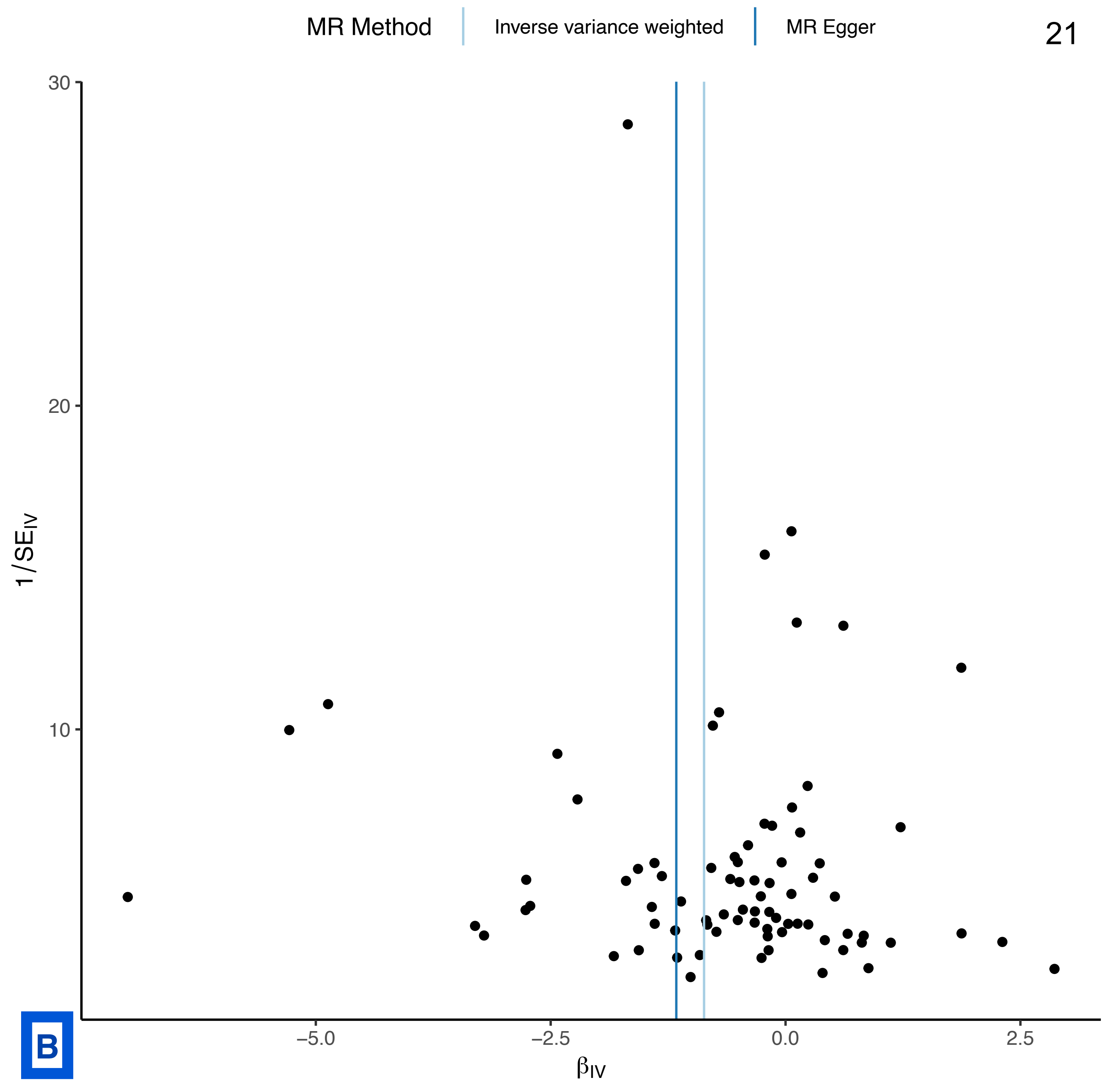
MR Method
Inverse variance weighted
MR Egger
30
20
10
1/SE$_{IV}$
−5.0
−2.5
0.0
2.5
β$_{IV}$
B

Supplemental File 6: Baseline characteristics for participants of observational study and descriptive statistics.

Table: Baseline characteristics for participants of observational study

**Characteristic**	**Healthy Participants,** N = 6,504[1]	**Participants with Pre-T2D**, N = 1,096[1]	**p-value**[2]
Sex			0.076
Female	3,147 (48%)	562 (51%)	
Male	3,357 (52%)	534 (49%)	
Age (years)	50 (41, 58)	56 (49, 61)	<0.001
HbA1c (%)	5.40 (5.20, 5.60)	6.10 (6.00, 6.20)	<0.001
Fasting Glucose (mmol/L)	5.10 (4.70, 5.50)	5.70 (5.20, 6.20)	<0.001
Triglyceride (mmol/L)	1.16 (0.85, 1.68)	1.33 (0.99, 1.92)	<0.001
HDL Cholesterol (mmol/L)	1.29 (1.07, 1.57)	1.19 (1.01, 1.43)	<0.001
Hemoglobin (g/L)	145 (136, 154)	144 (134, 153)	<0.001
Triglyceride Glucose Index	8.46 (8.12, 8.85)	8.71 (8.36, 9.08)	<0.001
Predicted A1c, adjusted for age, sex (%)	5.04 (4.97, 5.11)	5.14 (5.06, 5.23)	<0.001
Glycation Gap[3]	0.36 (0.12, 0.57)	1.01 (0.89, 1.13)	<0.001

[1] *Median (IQR); n (%)*

[2] *Wilcoxon rank sum test; Pearson's Chi-squared test*

[3] *Actual HbA1c – Predicted HbA1c*

Table: Linear Regression for Triglyceride Glucose Index (exposure) on Glycation Gap (outcome)*

**Population (n)**	**Beta**	**Standard error**	**y-intercept**	**p-value**
**Pre-T2D (1096)**	**-0.087**	**0.009**	**1.77**	**<0.0001**
**Healthy (6504)**	**0.023**	**0.007**	**0.14**	**<0.0001**
**HbA1c < 5% (681)**	**0.071**	**0.01**	**0.41**	**<0.0001**
**HbA1c 5 – 5.4% (2948)**	**0.047**	**0.005**	**0.6**	**<0.0001**
**HbA1c 5.5 – 5.9% (2875)**	**0.054**	**0.006**	**1.06**	**<0.0001**

[*]calculated as Actual HbA1c – Predicted HbA1c using model adjusted for age, sex (18)

Supplementary File 7

Link: https://www.dropbox.com/s/827zwgfwszefe36/SF8_FP_LOO_Export_All_Analyses.xlsx?dl=0
